# Protein kinase Pi65 regulates rice blast resistance through phosphorylation-dependent signaling and metabolic reprogramming

**DOI:** 10.3389/fgene.2025.1715247

**Published:** 2026-01-05

**Authors:** Lili Wang, Hongwei Chen, Xiaohang Zhou, Bowen Yan, Zhiqiang Tang, Zuobin Ma, Dianrong Ma, Wenjing Zheng

**Affiliations:** 1 Rice Research Institute of Liaoning Province, Liaoning Academy of Agricultural Sciences, Shenyang, China; 2 Rice Research Institute of Shenyang Agricultural University, Shenyang, China; 3 Liaoning Academy of Agricultural Sciences, Shenyang, China

**Keywords:** gene, phosphorylation, proteomics, rice, rice blast

## Abstract

Rice blast is a major fungal disease that threatens global rice production and is caused by the fungus *Magnaporthe oryzae*. Therefore, cloning rice blast resistance-related genes, conducting indepth analyses of the interaction mechanisms between *M. oryzae* and rice, elucidating rice disease resistance pathways, and developing new resistant germplasms are crucial for ensuring food security. This study took the rice blast resistance-related protein kinase Pi65 as the research object and explored its regulatory role in the immune response of rice through protein phosphorylation omics and protein interaction verification. The experimental results demonstrated that Pi65 exhibited autophosphorylation kinase activity. Based on phosphoproteomic analysis, 572 and 107 differentially regulated phosphoproteins (DPPs) were identified in Pi65-knockout (KO) and Pi65-overexpression (OE) lines, respectively, compared with the wild type (WT). These DPPs showed significant changes in signal transduction, metabolic processes, and subcellular localization, indicating that altered Pi65 expression affects phosphorylation homeostasis in rice leaves. KEGG and GO enrichment analyses revealed that the DPPs in KO lines were mainly associated with biological processes such as nitrogen cycling and non-homologous end joining, whereas DPPs in OE lines were significantly enriched in pathways related to the calvin cycle, glycolysis, and RNA binding. Thus, Pi65 may participate in the regulation of cellular metabolism by modulating nuclear phosphorylation networks and post-transcriptional modification processes. Protein interaction validation experiments further confirmed that Pi65 directly interacted with the redox regulatory protein OsAPX4 and the phosphate transporter OsPHF1, linking Pi65 function to redox homeostasis and phosphorus signaling. These findings suggest that Pi65 acts as a key regulatory hub that integrates biotic and abiotic stress signals to modulate rice blast resistance via phosphorylation-dependent signaling cascades. This study provides new insights into the roles of plant kinases in multi-stress responses and offers potential candidate targets for genetic improvement of crop stress resistance.

## 1 Introduction

Rice is a major global food crop, and its cultivation directly affects food security and consumer wellbeing (Yang et al., 2025). As a widespread disease caused by the pathogenic fungus *M. oryzae*, rice blast poses a serious threat to global rice production. It can cause substantial yield losses, typically ranging from 10% to 30%, and may even lead to complete crop failure during severe epidemics (Devanna et al., 2022; Yan and Talbot, 2016). Statistical data indicate that from 2012 to 2021, the annual average incidence area of rice blast in China reached 3.815 × 10^6^/ha, causing an average annual yield loss of approximately 3.68 42 × 10^5^/t of rice. (Wang et al., 2023). Owing to the high genetic variability of *Magnaporthe oryzae*, some disease-resistant rice varieties may lose their resistance within 3–5 years of deployment. Therefore, exploring gene resources with broad-spectrum and durable disease resistance is essential for sustainable control of rice blast.

Currently, more than 140 rice blast resistance (*R*) genes have been identified, of which over 30 have been cloned, including *Pizt*, *Pi9*, *Pi50*, *Pigm*, *Pi2*, *Pi5*, *Pi1*, *Pb1*, and *Pita* (Xiao et al., 2025). Most cloned rice blast R genes encode nucleotide-binding leucine-rich repeat (NLR) proteins, which typically recognize specific pathogen effectors directly or indirectly to activate immune responses (Ngou et al., 2022; Kourelis and Adachi, 2022; Li et al., 2020). NLR-type R genes are often present in the rice genome as tandem repeats, and most are located on chromosomes 6, 11, and 12. For instance, approximately 21 R genes are located on chromosome 6, including *Piz*, *Pi2*, *Pizt*, *Pi9*, *Pigm*, and *Pi50*, which are multiple alleles at the *Piz* locus. *Pi2* and *Pi9* confer high resistance to multiple physiological races of *M. oryzae* from diverse regions (Liu et al., 2002; Chen et al., 2001), whereas *PigmR* provides broad-spectrum resistance, and *PigmS* mitigates yield penalties associated with *PigmR*, thereby achieving a balance between resistance and productivity (Deng et al., 2017). Studies have reported that plant NLR proteins often function in synergistic pairs, categorized as sensor NLRs and helper NLRs. The sensor NLR recognizes pathogenic AVR effectors through a specialized domain, whereas the helper NLR activates downstream immune signaling. In the *Pikp* system, the immune receptor complex consists of the sensor NLR *Pikp-1* and the helper NLR *Pikp-2*. Pikp-1 protein contains a heavy metal-associated (HMA) domain that specifically binds to the *M. oryzae* effector Avr-PikD. *Pikp*-1 and *Pikp*-2 mediate resistance to *M. oryzae* races carrying Avr-PikD (Maqbool et al., 2015). The variation in the amino acid sequence of the HMA domain directly determines the recognition specificity of rice NLRs for AVR effectors. Different alleles of the rice resistance gene *Pik* (including *Pikm*, *Piks*, *Pikp*, and *Pikh*) can recognize distinct Avr-Pik effector variants, conferring race-specific resistance to *M. oryzae* (Yoshida et al., 2009). Approximately 27 resistance genes are located on chromosome 12, including *Pita* and *Pita2*. Recent studies have indicated that resistance mediated by *Pita* is determined by allelic variations in *Ptr*. The *PtrA* allele recognizes all natural *AVR*-*Pita* variants and confers *Pita2* resistance, whereas the *PtrB* allele recognizes only specific *AVR*-*Pita* variants, conferring *Pita* resistance (Xiao et al., 2024).

In addition to typical NLR-type disease resistance genes, some atypical NLR genes encode protein kinases and regulatory factors. *Pid2* encodes a receptor-like kinase (RLK) containing a signal peptide, a B-lectin domain, a PAN domain, a transmembrane domain, and a serine/threonine kinase (STK) domain (Chen et al., 2006). Another example is Bsr-d1, which encodes a C2H2-type transcription factor that promotes the expression of peroxidase genes and reduces reactive oxygen species (ROS) accumulation, rendering rice susceptible to diseases. However, the bsr-d1 mutant increases the ROS levels and confers broad-spectrum resistance (Li et al., 2017). The proteasome maturation factor gene UMP1 has a naturally occurring allele UMP1R2115, which increases proteasome content and catalytic activity, accelerates the degradation of peroxidases and catalases, leads to ROS accumulation, and enhances broad-spectrum resistance to *M. oryzae* (Hu et al., 2023).

Protein phosphorylation is a common regulatory mechanism in cellular signal transduction networks. In the rice immune response against *M. oryzae*, the activation of immune signaling pathways largely depends on the phosphorylation status of key regulatory proteins. RLKs and receptor-like cytoplasmic kinases (RLCKs) are critical regulators that confer broad-spectrum resistance to rice blast (Mao et al., 2022). BSR1 is a representative RLCK family protein, and its overexpression in rice enhances resistance to multiple pathogens, such as *M. oryzae* and *Xanthomonas oryzae pv. oryzae* (Maeda et al., 2016). The RLK protein SDS2 transmits phosphorylation signals through RLCK118, regulates the activity of NADPH oxidase OsRbohB, promotes ROS accumulation, and thereby enhances the ability of rice to resist blast disease (Fan et al., 2018). During immune signal transduction, RLKs and RLCKs frequently form functionally coordinated protein complexes, such as BAK1–BIK1 and OsSERK1–OsRLCK185, which play key roles in relaying extracellular signals to intracellular responses (Lin et al., 2014; Wang et al., 2017).

In the early stage of this study, a rice blast resistance gene, *Pi65(t)*, was mapped from the *Japonica* rice cultivar Gangyu 129 (GY129) using Super-BSA technology and was located between 30.2 and 31.2 Mb on chromosome 11. By constructing a backcross population, screening recombinants, and evaluating their phenotypes, *Pi65(t)* was finely mapped to the region of Chr11:30.57–30.63 Mb, which contained four candidate genes (Zheng et al., 2016). The disease resistance function of Pi65 was subsequently validated through gene knockout and overexpression analyses, showing that Pi65 encodes a leucine-rich repeat receptor-like protein kinase. Transcriptome sequencing revealed that Pi65 conferred resistance to *M. oryzae* by modulating photosynthesis and primary metabolic processes (Wang et al., 2022). To further investigate whether Pi65 possesses kinase activity and how it integrates various pathways to regulate resistance to rice blast, this study built upon previous work by detecting the kinase activity of Pi65. Previous studies confirmed the kinase activity of Pi65 and demonstrated its autophosphorylation capability. Phosphoproteomic analysis of wild-type GY129 and Pi65 knockout and overexpression lines revealed that Pi65 influenced signal transduction, metabolic pathways, and organelle-related protein distribution via phosphorylation-level regulation. Additionally, Pi65 was found to interact with OsAPX4 and OsPHF1, linking its function to redox homeostasis and phosphorus signaling. These findings suggest that Pi65 acts as a key regulatory node that integrates environmental signals and modulates rice disease resistance via phosphorylation-mediated signaling cascades. By verifying Pi65’s kinase activity, its phosphorylation-regulated pathways, and its protein-protein interactions, this study provides deeper insights into how Pi65 confers blast resistance in rice. This research will offer robust support for molecular breeding of blast-resistant japonica rice in northern China.

## 2 Materials and methods

### 2.1 Plant materials

The *Japonica* rice variety GY129 is resistant to most tested *M. oryzae* isolates from Liaoning Province, China, and has been widely used in international research and breeding (Zheng et al., 2016; Mukhina et al., 2020). The plant materials used in this study included wild-type GY129 and Pi65 knockout (KO) mutants generated in the GY129 genetic background (Wang et al., 2022).

The full-length CDS of Pi65 was amplified using primers listed in Supplementary Table S1, and the amplified fragment was cloned into the pCambia1301-UbiN vector at the BamHI restriction site to generate the overexpression construct pCambia1301-UbiN-OsPi65 (OE). The recombinant vector was introduced into the calli of *Japonica* rice GY129 via *Agrobacterium tumefaciens*-mediated transformation. Transgenic plants were selected on a medium containing 300 mg/L carbenicillin and 50 mg/L hygromycin, and the presence of the hygromycin resistance gene was verified using PCR. All transgenic plants were grown under controlled conditions in an artificial climate chamber at the Liaoning Academy of Agricultural Sciences in Shenyang, China. More than 10 transgenic lines were obtained, and three independent T_2_ lines were selected for further analyses.

All rice seeds were sown in black plastic containers (10 cm × 7.0 cm × 8.5 cm) filled with sterilized seedling substrate. The containers were placed in blue water trays filled to one-third of their volume (34.5 cm × 47 cm × 15 cm). The seedlings were grown in a greenhouse at 25 °C–30 °C under a 16 h light/8 h dark photoperiod until reaching the 3.5-leaf stage (approximately 3 weeks). At this stage, leaf samples were collected, rapidly frozen in liquid nitrogen, and stored at −80 °C for subsequent protein phosphoproteomic analysis.

### 2.2 *In Vitro* phosphorylation assay

Recombinant GST-tagged Pi65 and GST control proteins were expressed in *Escherichia coli* BL21(DE3) induced with 0.5 mM IPTG at 16 °C for 16 h and purified using glutathione Sepharose 4B beads. Purified proteins (1 μg per reaction) were incubated in kinase buffer (50 mM Tris-HCl pH 7.5, 10 mM MgCl_2_, 1 mM DTT, and 500 μM ATP) at 30 °C for 60 min to allow autophosphorylation. Parallel samples were treated with 400 U λ phosphatase (New England Biolabs, Lot:10117443) at 30 °C for 30 min prior to analysis. The samples were separated by 10% SDS-PAGE and transferred to PVDF membranes for immunoblotting using an anti-phospho-threonine/tyrosine antibody (Cell Signaling Technology, Lot: 9381, diluted 1:2000 in 5% BSA/TBST), followed by incubation with an HRP-conjugated secondary antibody and ECL detection. Equivalent protein loading was verified using Coomassie Brilliant Blue-stained replicate gels.

### 2.3 Phosphoproteomic analysis

#### 2.3.1 Sample preparation

To minimize the impact of random transgene insertion events on phosphoproteomic analysis, three independent overexpression lines were pooled to generate a composite sample, which was subsequently subjected to phosphoproteomic profiling with three technical replicates. Protein extraction was performed using a phenol-based lysis buffer (Sigma-Aldrich/Thermo Scientific inhibitors) after cryogenic grinding of the rice tissues. After methanol/acetone precipitation and BCA quantification (Beyotime Lot: P0012S), the proteins were reduced with 5 mM TCEP (37 °C for 30 min), alkylated with 15 mM iodoacetamide (room temperature for 45 min in the dark), and digested overnight with trypsin (Promega, 1:100 w/w, 37 °C). The resulting peptides were desalted on C18 columns, and phosphopeptides were enriched using High-Select Fe-NTA kits (Thermo Scientific, Lot: A32992). The enriched samples were lyophilized before LC-MS/MS analysis.

#### 2.3.2 NanoLC-MS/MS analysis

Approximately 200 ng of phosphopeptides per sample were separated using a nanoElute2 UPLC system (Bruker) equipped with a PepSep C18 column (1.9 μm, 75 μm × 15 cm). Chromatographic separation was performed using a 44 min gradient with 0.1% formic acid in water (mobile phase A) and 0.1% formic acid in acetonitrile (mobile phase B). The eluted peptides were analyzed using a timsTOF Pro 2 mass spectrometer (Bruker) operated in the DDA-PASEF mode (m/z 100–1700). Collision energies were adjusted from 20 to 59 eV based on the ion mobility (1/K_0_ = 0.6–1.6 Vs/cm^2^).

#### 2.3.3 Data analysis

Raw data were processed using SpectroMine (v4.2.230428.52329; Biognosys) with the Pulsar search engine against the UniProt *Oryza sativa* database (39,947 entries, 2025–03–05). The search parameters included trypsin digestion (maximum two missed cleavages), carbamidomethylation (C, fixed modification), and variable modifications for oxidation (M), phosphorylation (S/T/Y), and acetylation (N-terminus). Mass tolerances were set at 20 ppm for MS1 and MS2. The false discovery rate was set at ≤1% for both PSM and peptide levels. Label-free quantification employed reporter intensity correction with median normalization across samples. The mass spectrometry proteomics data has been deposited to the ProteomeXchange.Consortium (https://proteomecentral.proteomexchange.org) via the iProX partner repository (Ma et al., 2019; Chen et al., 2021) with the dataset identifier PXD071994.

#### 2.3.4 Bioinformatic analysis

Functional annotation of phosphorylated proteins was performed using Gene Ontology (GO) via InterProScan (v5.14-53.0). Kyoto Encyclopedia of Genes and Genomes (KEGG) pathway enrichment analysis was conducted using the KEGG database portal. Protein–protein interaction networks were generated using the STRING database and visualized in Cytoscape (v3.8.2). Subcellular localization was predicted using WoLF PSORT (v0.2).

### 2.4 Yeast two-hybrid assay

Yeast two-hybrid (Y2H) screening was performed to identify Pi65-interacting proteins. The bait construct pGBKT7-Pi65 (Pi65 fused to the GAL4 DNA-binding domain) was transformed into the Y2HGold strain using the PEG/LiAc method. The absence of autoactivation was confirmed by no growth on SD/-Leu/-Trp/-His/-Ade plates when co-transformed with the empty pGADT7 vector. The bait strain was then mated with a rice cDNA library constructed from *M. oryzae*-infected *O*. *sativa* ssp. *Japonica* cv. GY129 leaves, which were pre-transformed into Y2HGold. Diploid cells were selected on SD/-Leu/-Trp plates and subsequently screened on SD/-Leu/-Trp/-His/-Ade/X-α-Gal plates. Blue colonies appearing after 5–7 days were isolated, and prey plasmids were recovered using the Yeast Plasmid Kit (Zymo Research, Lot: D2002) and sequenced. For pairwise verification, candidate genes (e.g., *LOC_Os08g43560* and *LOC_Os07g09000*) were cloned into pGADT7 and co-transformed with pGBKT7-Pi65 into Y2HGold, and interactions were assessed based on growth on selective media.

### 2.5 NanoLuc luciferase complementation assay (ncLUC)

The target proteins were fused to complementary fragments of NanoLuc luciferase (Promega NanoBiT System) and cloned into plant expression vectors under the control of the CaMV 35S promoter. *Agrobacterium tumefaciens* strain GV3101 carrying the constructs was co-infiltrated into young *Nicotiana benthamiana* leaves using an infiltration buffer (10 mM MgCl_2_, 10 mM MES pH 5.6, and 200 μM acetosyringone). After 48–72 h of incubation at 22 °C under a 16 h light/8 h dark photoperiod, luciferase activity was detected by applying Nano-Glo Luciferase Assay Reagent with furimazine substrate (Promega) directly to the leaf surfaces. Whole-leaf luminescence was captured using an *in vivo* imaging system (IVIS Spectrum, PerkinElmer or equivalent) with a 1-min exposure time. Signal quantification was performed after background subtraction using negative co-infiltration controls (empty vectors).

### 2.6 Co-immunoprecipitation

Co-immunoprecipitation (Co-IP) assays were performed on rice protoplasts. Protoplasts were isolated from 10-day-old *O*. *sativa* ssp. *japonica* seedlings via enzymatic digestion (1.5% cellulase R10% and 0.3% macerozyme R10) and transfected with GFP- or HA-tagged constructs using PEG-mediated transfection. After 12–16 h of incubation in W5 solution (22 °C, dim light), the cells were lysed in IP buffer (50 mM Tris-HCl, pH 7.5; 150 mM NaCl; 1% Triton X-100) containing protease inhibitors. Lysates were incubated with anti-GFP (Roche, Lot: 11814460001) or anti-HA (Abcam, Lot: Ab9110) antibodies for 2 h at 4 °C, followed by incubation with Protein A/G magnetic beads (Yeasen, Lot: 36403ES08) for 1 h. The beads were washed four times and resuspended in 2× SDS loading buffer. The immunoprecipitated proteins were separated by SDS-PAGE and detected by immunoblotting.

## 3 Results

### 3.1 Pi65 exhibits kinase activity via autophosphorylation

**FIGURE 1:**
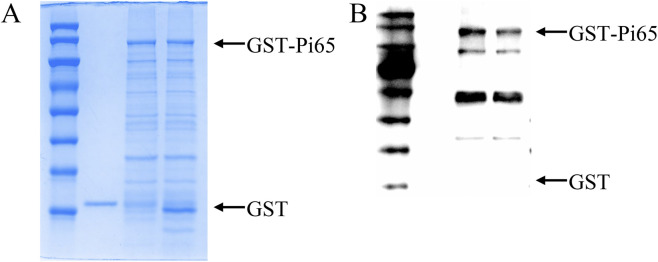
*In vitro* phosphorylation activity of Pi65. **(A)** Coomassie Brilliant Blue staining of purified recombinant proteins. **(B)** Immunoblot analysis of phosphorylation levels.

Immunoblot analysis using an anti-phospho-threonine/tyrosine antibody under automated exposure conditions revealed no detectable signal in the GST control (Lane 2), whereas untreated GST-Pi65 (Lane 3) exhibited a strong phosphorylation signal. Notably, λ-phosphatase treatment of GST-Pi65 (Lane 4) caused a near-complete loss of the phosphorylation signal, whereas the molecular weight of GST-Pi65 remained unchanged, confirming specific dephosphorylation (Figure 1). These results demonstrated that Pi65 possessed intrinsic autophosphorylation kinase activity.

### 3.2 Phosphoprotein identification in rice leaves

**FIGURE 2:**
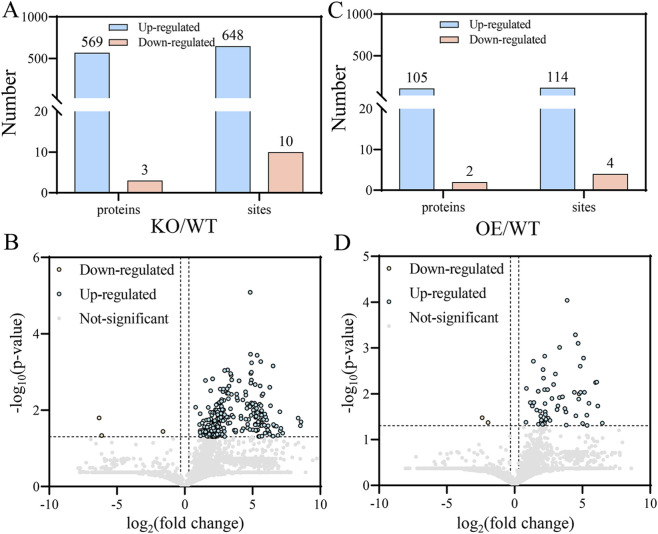
Differential regulation of phosphorylated proteins and phosphorylation sites in rice leaves. **(A)** Number of up- and downregulated phosphorylated proteins and phosphorylation sites in rice leaves between KO and WT. **(B)** Volcano plot of phosphosite changes between KO and WT. **(C)** Numbers of up- and downregulated phosphorylated proteins and phosphorylation sites in rice leaves between OE and WT. **(D)** Volcano plot of phosphosite changes between OE and WT. WT, wild-type; KO, knockout line; OE, over expression line.

A comprehensive phosphoproteomic analysis identified 13,466 phosphorylated proteins with 15,469 phosphorylation sites in all rice leaf samples. Compared with WT, the KO line exhibited 648 significantly upregulated and 10 downregulated phosphorylation sites (fold change >1.5, p < 0.05), corresponding to 569 upregulated and three downregulated differentially regulated phosphoproteins (DPPs) (Figures 2A,B). Notably, 21 DPPs associated with environmental stress responses were identified. In contrast, the OE line exhibited 114 upregulated and four downregulated phosphorylation sites, corresponding to 105 upregulated and two downregulated DPPs (Figures 2C,D).

### 3.3 Functional annotation and subcellular localization of DPPs

**FIGURE 3:**
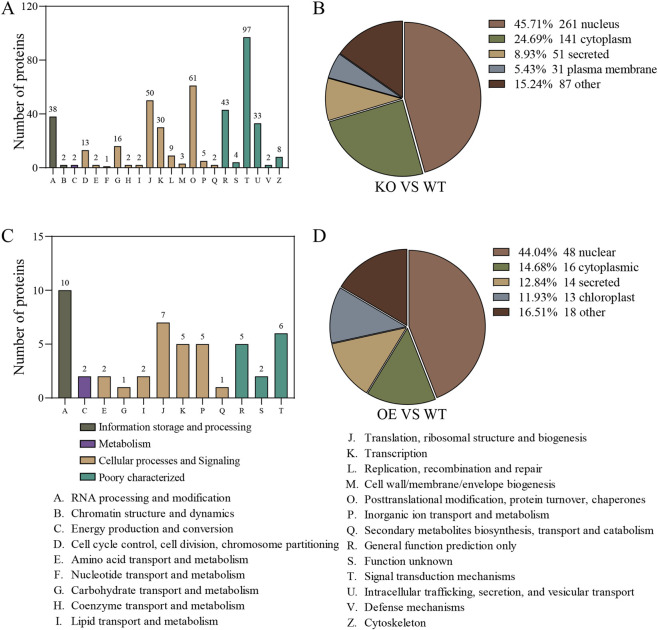
Functional classification of phosphorylated proteins (DPPs). **(A,C)** COG/KOG functional classification of DPPs identified in KO vs. WT **(A)** and OE vs. WT **(C)** comparisons. **(B,D)** Distribution of DPPs among major functional categories for KO vs. WT **(B)** and OE vs. WT **(D)**. WT, wild-type; KO, knockout line; OE, over expression line.

In the KO line, DPPs were primarily involved in processes related to signal transduction, post-translational modification/chaperone activity, translation machinery, intracellular trafficking, and carbohydrate metabolism. These proteins were predominantly localized to the nucleus (45.71%), with increased cytoplasmic representation (24.69%) and significant distribution in secretory pathways (8.93%) and plasma membrane-associated compartments (5.43%) (Figures 3A,B). In the OE line, DPPs were assigned to 12 functional categories, with enrichment in RNA processing and modification, translation/ribosomal biogenesis, signal transduction, transcription, and inorganic ion transport. Subcellular localization analysis revealed nuclear enrichment (44.04%), along with substantial cytoplasmic (14.68%), secreted (12.84%), and chloroplast (11.93%) localization (Figures 3C,D).

### 3.4 GO and KEGG enrichment analysis

**FIGURE 4:**
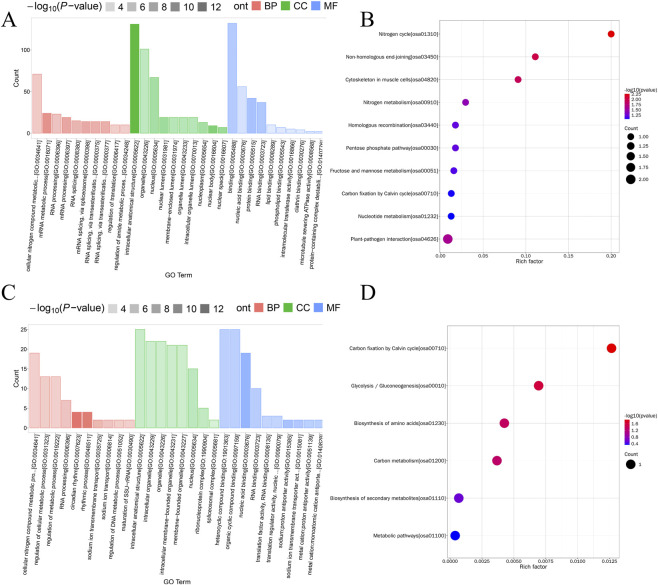
GO and KEGG pathway enrichment analyses of differentially phosphorylated proteins (DPPs) in phosphoproteomic data. **(A)** GO enrichment analysis of DPPs in the Pi65 KO line across biological process, molecular function, and cellular component categories. **(B)** GO enrichment analysis of DPPs in the Pi65 OE line across biological process, molecular function, and cellular component categories. **(C)** KEGG pathway enrichment of DPPs in the Pi65 KO line. **(D)** KEGG pathway enrichment of DPPs in the Pi65 OE line. WT, wild-type; KO, knockout line; OE, overexpression line.

GO enrichment analysis revealed distinct patterns between genotypes. In the KO line, DPPs were enriched in intracellular membrane-bound organelles for cellular component categories, whereas molecular function categories were dominated by protein binding and nucleic acid binding. The enriched biological processes were associated with cellular metabolic processes and nitrogen compound metabolism (Figure 4A). In contrast, DPPs in the OE line demonstrated enrichment in RNA binding and cellular biosynthetic and nitrogen metabolism pathways (Figure 4B). KEGG enrichment analysis further demonstrated that DPPs in the KO line were significantly enriched in nitrogen cycle, non-homologous end joining, and cytoskeletal regulation pathways (Figure 4C), whereas DPPs in the OE line were significantly enriched in carbon fixation through the Calvin cycle and glycolysis/gluconeogenesis pathways (Figure 4D).

### 3.5 Identification of Pi65-Interacting proteins by yeast two-hybrid screening

A rice cDNA library was screened using Pi65 as bait to identify its interacting partners. A total of 100 candidate clones exhibited growth on high-stringency SD/-Leu/-Trp/-His/-Ade plates, indicating a potential interaction with Pi65. Sequencing and BLAST analyses against the Rice Genome Annotation Project (RGAP) database revealed that these genes encoded proteins involved in diverse biological processes, including redox regulation, phosphate transport, and signal transduction. Among these, OsAPx4 (LOC_Os08g43560), which encodes an ascorbate peroxidase, and OsPHF1 (LOC_Os07g09000), which encodes a phosphate transporter trafficking factor, were selected for further validation because of their reported roles in stress responses (Supplementary Table S2).

### 3.6 Validation of the interaction between Pi65 and OsAPx4

**FIGURE 5:**
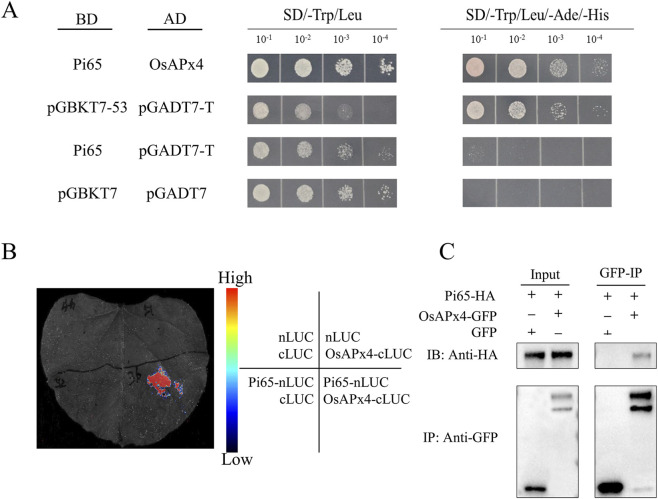
Validation of the physical interaction between Pi65 and OsAPx4. **(A)** Yeast two-hybrid assay performed in the strain Y2HGold revealed a specific interaction between BD-Pi65 and AD-OsAPx4, with pGBKT7-53 + pGADT7-T serving as a positive control and pGBKT7-Pi65 + pGADT7 or pGBKT7 + pGADT7-OsAPx4 as negative controls. **(B)** Co-immunoprecipitation in *Nicotiana benthamiana* confirmed the interaction, where Pi65-HA specifically co-precipitated with OsAPx4-GFP using anti-GFP beads, as detected by anti-HA immunoblotting. Controls included OsAPx4-GFP alone and Pi65-HA co-expressed with free GFP. **(C)** Bimolecular fluorescence complementation assay demonstrated luminescence signal in leaves co-infiltrated with Pi65-nLUC and OsAPx4-cLUC constructs.

The yeast two-hybrid assay confirmed a specific interaction between the bait protein Pi65 (pGBKT7-Pi65) and the prey protein OsAPx4 (pGADT7-OsAPx4), as evidenced by robust colony growth on SD/-Leu/-Trp/-His/-Ade plates (Figure 5A). To evaluate this interaction in planta, a NanoLuc luciferase complementation assay in *Nicotiana benthamiana* leaves indicated that the co-expression of Pi65-nLUC and OsAPx4-cLUC reconstituted luciferase activity, producing luminescence signals 12.3-fold higher than those of the negative controls (empty nLUC/cLUC vectors or non-interacting pairs), with luminescence localized to the co-infiltration zones (Figure 5B). Further validation in rice protoplasts using Co-IP demonstrated that GFP-tagged OsAPx4 specifically co-precipitated with HA-tagged Pi65 using an anti-HA antibody, whereas no interaction was detected in single-tagged or vector-only controls (Figure 5C). As OsAPx4 encodes an ascorbate peroxidase, its interaction with Pi65 suggests a regulatory role for Pi65 in redox homeostasis, consistent with the involvement of OsAPx4 in rice blast resistance.

### 3.7 Validation of the interaction between Pi65 and OsPHF1

**FIGURE 6:**
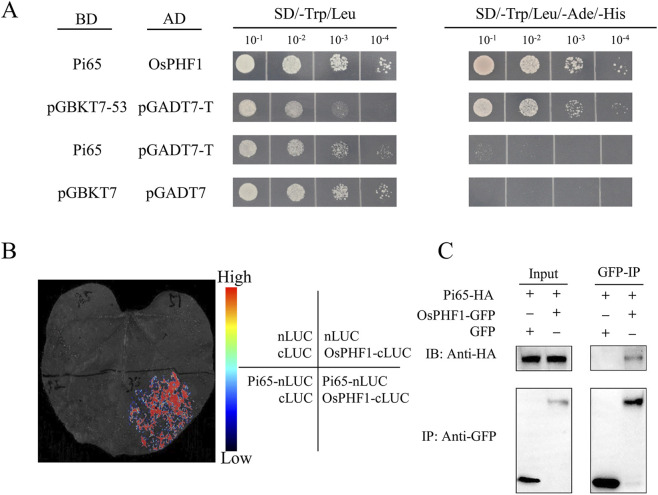
Validation of the physical interaction between Pi65 and OsPHF1. **(A)** Yeast two-hybrid assay performed in the strain Y2HGold revealed a specific interaction between BD-Pi65 and AD-OsPHF1, with pGBKT7-53 + pGADT7-T serving as a positive control and pGBKT7-Pi65 + pGADT7 or pGBKT7 + pGADT7-OsPHF1 as negative controls. **(B)** Co-immunoprecipitation in *Nicotiana benthamiana* confirmed the interaction, where Pi65-HA specifically co-precipitated with OsPHF1-GFP using anti-GFP beads, as detected by anti-HA immunoblotting. Controls included OsPHF1-GFP alone and Pi65-HA co-expressed with free GFP. **(C)** Bimolecular fluorescence complementation assay demonstrated luminescence signal in leaves co-infiltrated with Pi65-nLUC and OsPHF1-cLUC constructs.

The interaction between Pi65 and OsPHF1 was confirmed using multiple experimental systems. In the yeast two-hybrid assay, co-expression of Pi65 (pGBKT7-Pi65) and OsPHF1 (pGADT7-OsPHF1) resulted in robust colony growth on SD/-Leu/-Trp/-His/-Ade plates, whereas the control combinations failed to grow, indicating interaction specificity (Figure 6A). In *N. benthamiana* leaves, split luciferase complementation analysis revealed strong luminescence signals following the co-expression of Pi65-nLUC and OsPHF1-cLUC, whereas the negative controls (nLUC/cLUC empty vectors or non-interacting pairs) showed no detectable signals (Figure 6B). Co-IP assays in rice protoplasts transiently expressing Pi65-HA and OsPHF1-GFP further confirmed their physical association, as OsPHF1-GFP specifically co-precipitated with Pi65-HA using an anti-HA antibody, with no interaction observed in the single-tag or vector-only controls (Figure 6C). Collectively, these results provide strong evidence for the specificity of the Pi65–OsPHF1 interaction.

## 4 Discussion

As a major plant pathogen threatening global food security, *M. oryzae* causes rice blast, one of the most destructive rice diseases, causing severe yield losses. Although NLR-type genes directly confer resistance to rice blast, other genes contribute to the regulation of this process through diverse molecular mechanisms. A representative example is the interaction between OsCERK1 and OsCIE1, where the coordinated regulation of their phosphorylation and ubiquitination states collectively modulate disease resistance (Wang G. et al., 2024).

### 4.1 Pi65 regulates rice blast resistance through phosphorylation-dependent nitrogen and energy metabolism

In this study, we confirmed that Pi65 possesses autophosphorylation kinase activity and that λ-phosphatase treatment substantially eliminated its phosphorylation signal, demonstrating its phosphorylation-dependent catalytic activity. Phosphoproteomic analysis revealed 658 differential phosphorylation sites in the KO line, corresponding to 572 DPPs. Among these DPPs, in the Pi65 overexpression (OE) line, 118 differentially phosphorylated sites were identified, corresponding to 107 differentially regulated phosphoproteins. These results suggest that Pi65 knockout may trigger immune compensation by relieving the negative regulation of multiple R proteins and signaling modules, whereas Pi65 overexpression may enhance resistance by increasing the abundance of resistance-associated effector proteins. DPPs in the KO line were enriched in signal transduction, post-translational modification, and carbohydrate metabolism, with subcellular localization predominantly in the nucleus (45.71%) and cytoplasm. In contrast, DPPs in the OE line were enriched in RNA processing, ribosome biogenesis, and inorganic ion transport, with 44.04% localized in the nucleus. These findings indicate that Pi65 participates in gene expression or signal transduction by regulating nuclear phosphorylation networks and that changes in its kinase activity significantly shift phosphorylation homeostasis in rice cells. Previous studies have shown that optimal fertilization strategies, particularly those that adjust nitrogen forms (favoring NH_4_^+^) and concentration, can balance rice growth and disease resistance, thereby reducing both disease incidence and pesticide use (Wang S. Y. et al., 2024).

Other studies have demonstrated that APIP5 exhibits RNA-binding activity and binds the mRNAs of two rice genes involved in disease resistance and cell death, thereby regulating basal immunity at the post-transcriptional level (Wang et al., 2016; Zhang et al., 2022). Similarly, activated OsMPK5 phosphorylates OsDRB1.4 to enhance its RNA-binding ability and suppress resistance (Chen et al., 2025; Kankanala et al., 2007; Bolton et al., 2008; Kadota et al., 2015). To further clarify the functional roles of Pi65, GO functional classification and KEGG enrichment analyses were performed. In both KO and OE lines, DPPs were enriched in biological processes, cellular components, and molecular functions associated with cellular metabolism, nitrogen compound metabolism, membrane-bound organelles, and nucleic acid binding. In this study, the enhanced RNA-binding function and nitrogen metabolic pathways observed in the OE line suggest that Pi65, with phosphorylation modification as the core regulatory mechanism, mediates metabolic stability and immune balance by coordinating metabolic proteins, membrane signaling proteins, and nucleic acid–binding proteins.

The activation and maintenance of rice immune responses against *M. oryzae* require substantial energy. Upon PAMP recognition, rice cells initiate a series of signaling processes, including calcium ion fluctuations and rapid ROS generation, all of which rely on energy supply (Kankanala et al., 2007; Bolton et al., 2008; Kadota et al., 2015). The Calvin cycle and glycolysis provide carbon skeletons and substrates for the synthesis of defense-related compounds. Phenylalanine ammonia-lyase (PAL), a key rate-limiting enzyme in the phenylpropanoid pathway, regulates the initial reactions of this metabolic route, producing compounds critical for disease resistance. Among these, lignin significantly enhances cell wall mechanical strength, forming an effective physical barrier against pathogen invasion (Xu et al., 2025). KEGG enrichment analysis in this study revealed that DPPs in Pi65-KO lines were significantly enriched in pathways related to nitrogen cycling and non-homologous end joining, whereas those in OE lines were enriched in the Calvin cycle and glycolysis/gluconeogenesis. In the absence of Pi65, alterations in its kinase activity may impair the balance between nitrogen-containing compound metabolism and immune metabolism, leading to metabolic disruption. Conversely, Pi65 overexpression may direct the enrichment of energy metabolism pathways toward the Calvin cycle and glycolysis by regulating its own expression level and providing energy for rice immune responses. This could support disease resistance-related physiological processes and enable positive regulation of rice blast resistance.

### 4.2 Pi65 orchestrates rice blast resistance by synergistically integrating ROS signaling and phosphate metabolism pathways

Yeast two-hybrid screening and related validation experiments confirmed that Pi65 directly interacted with the redox regulatory protein OsAPx4 and the phosphate transporter factor OsPHF1. Hydrogen peroxide is involved in the expression of ascorbate peroxidase and glutathione reductase in rice seedling leaves under heat shock and cadmium stress (Chou et al., 2012). OsAPx4 plays an important role in ROS-mediated leaf senescence (Ribeiro et al., 2017). The direct interaction between Pi65 and OsAPx4 may achieve dual regulation by stabilizing OsAPx4 function. The first is maintaining ROS levels within a safe range to avoid cellular damage caused by excessive ROS during *M. oryzae* infection while retaining sufficient ROS to activate downstream immune signaling. The second is the prevention of pathogen-induced premature senescence, thereby enhancing leaf resistance, maintaining normal leaf physiological functions, and reducing damage caused by *M. oryzae*.

OsPHF1 plays a key role in regulating the plasma membrane localization of low- and high-affinity phosphate (Pi) transporters in rice and determines Pi absorption and transport efficiency (Chen et al., 2011; Chen et al., 2015). The interaction between Pi65 and OsPHF1 may contribute to immunity by improving phosphorus absorption and distribution, thereby ensuring adequate phosphorus resources for synthesizing defense-related compounds during *M. oryzae* infection. This interaction can promote plant growth potential by improving phosphorus storage capacity and indirectly enhancing resistance to *M. oryzae*. ROS signaling and phosphorus metabolism are not independent pathways. Phosphorus metabolism provides raw materials for the synthesis of ROS-related enzymes, whereas ROS signaling regulates the expression of phosphorus transport-related genes. As a key regulatory protein that interacts with both OsAPx4 and OsPHF1, Pi65 may integrate these two pathways by maintaining ROS signaling homeostasis via OsAPx4 and ensuring phosphorus supply through OsPHF1 to support the synthesis of immune compounds. Ultimately, a coordinated regulatory network is established to enhance rice resistance to *M. oryzae*.

## 5 Conclusion

Pi65 is a kinase with autophosphorylation activity that dynamically regulates rice immunity through reversible phosphorylation. Phosphoproteomic analysis demonstrated that KO or OE of Pi65 altered cellular phosphorylation homeostasis, influencing signal transduction, post-translational modification, and metabolic processes. In the OE line, Pi65 may enhance the post-transcriptional regulation of disease resistance genes by promoting RNA processing and related pathways. In contrast, the KO line exhibited weakened immunity owing to disruptions in signaling and carbon metabolism. KEGG analysis indicated that the Calvin cycle and glycolysis were enriched in the OE line, suggesting that Pi65 may guide energy metabolism to supply carbon skeletons and ATP required for synthesizing disease resistance-related compounds, thereby strengthening the physical defense of cell walls.

Pi65 formed a synergistic regulatory network through direct interactions with OsAPx4 and OsPHF1. First, Pi65 stabilized the activity of OsAPx4 and maintained the dynamic balance of ROS during *M. oryzae* infection, ensuring both cellular protection and activation of defense-related genes. Second, its interaction with OsPHF1 optimized phosphorus uptake and distribution, providing sufficient substrates for the synthesis of disease resistance-related molecules. Enhanced phosphorus metabolism may further promote plant growth vigor, thereby indirectly improving disease resistance. In addition, ROS and phosphorus metabolism were intrinsically interconnected. Phosphorus served as a substrate for ROS-related enzymes, whereas ROS signaling regulated the expression of phosphorus transporter genes. Pi65 integrated these two pathways to establish a synergistic regulatory network, collectively enhancing rice resistance to *M. oryzae*. In summary, Pi65 strengthens the rice immune defense system through phosphorylation-dependent signal regulation, reprogramming of energy metabolism, and coordinated interaction of ROS and phosphorus metabolic pathways mediated by its interacting proteins. These findings provide new insights into how plant kinases orchestrate multidimensional stress responses and suggest potential targets for breeding stress-resistant crops.

## Data Availability

The data presented in the study are publicly available. This data can be found in the ProteomeXchange repository at http://proteomecentral.proteomexchange.org/cgi/GetDataset?ID=PXD071994, with the accession number PXD071994.
