# Supplementary material for: Protein kinase Pi65 regulates rice blast resistance through phosphorylation-dependent signaling and metabolic reprogramming
Source: Front Genet. 2026 Jan 5;16:1715247. doi: 10.3389/fgene.2025.1715247 (PMC12812394; doi:10.3389/fgene.2025.1715247)
Supplement: Supplementary file 1 [file Table1.docx]

| Table S1 Detection of hygromycin in overexpressed lines | | | | | | | | |
| --- | --- | --- | --- | --- | --- | --- | --- | --- |
| Gene | | | Primer | Primer sequence | | Purpose | | |
| *Pi65* | | | F | ATGGCGTTTAGAATGCCAGTACGGA | | Construction of Pi65 overexpression vector | | |
|  | | | R | TCATTCTTTCGTATATGCAGGCAGGGCCAC | |  | | |
| Hyg | | | F | ACGGTGTCGTCCATCACAGTTTGCC | | Positive identification | | |
|  | | | R | TTCCGGAAGTGCTTGACATTGGGGA | |  | | |
| pGBKT7-*Pi65* | | | F | atggccatggaggccgaattcATGGCGTTTAGAATGCCAGTACGGA | | Yeast two-hybrid assay | | |
|  | | | R | cgctgcaggtcgacggatccTTCTTTCGTATATGCAGGCAGGGCCAC | |  | | |
| pGADT7-*OsAPx4* | | | F | gccatggaggccagtgaattcATGGCCGCCCCGGTCGTGGAC | | Yeast two-hybrid assay | | |
|  | | | R | cagctcgagctcgatggatcccTTACTTGCTCTTCTTAGAAGCC | |  | | |
| pGADT7-*OsPHF1* | | | F | gccatggaggccagtgaattcATGGCAGGCGGCGGAGGTGGCGA | | Yeast two-hybrid assay | | |
|  | | | R | cagctcgagctcgatggatcccTCACCAGGGGTTCTGGTCCTCA | |  | | |
| *Pi65*-nLUC | | | F | acgggggacgagctcggtaccATGGCGTTTAGAATGCCAGTACG | | Luciferase complementation assay | | |
|  | | | R | cgcgtacgagatctggtcgacTTCTTTCGTATATGCAGGCAGGGCCAC | |  | | |
| *OsAPx4*-cLUC | | | R | tacgcgtcccggggcggtaccATGGCCGCCCCGGTCGTGGAC | | Luciferase complementation assay | | |
|  | | | F | tgtagtccatttgttggatccTTACTTGCTCTTCTTAGAAGCC | |  | | |
| *OsPHF1*-cLUC | | | R | tacgcgtcccggggcggtaccATGGCAGGCGGCGGAGGTGGCGA | | Luciferase complementation assay | | |
|  | | | F | tgtagtccatttgttggatccTCACCAGGGGTTCTGGTCCTCA | |  | | |
| *Pi65*-HA | | | R | tatccagatccagtgggatccATGGCGTTTAGAATGCCAGTACGGA | | Co-immunoprecipitation | | |
|  | | | R | agtaagcttggtaccgagctcTTCTTTCGTATATGCAGGCAGGGCCAC | |  | | |
| *OsAPx4*-GFP | | | F | tatccagatccagtgggatccATGGCCGCCCCGGTCGTGGAC | | Co-immunoprecipitation | | |
|  | | | R | agtaagcttggtaccgagctcCTTGCTCTTCTTAGAAGCC | |  | | |
| *OsPHF1*-GFP | | | F | tatccagatccagtgggatccATGGCAGGCGGCGGAGGTGGCGA | | Co-immunoprecipitation | | |
|  | | | R | agtaagcttggtaccgagctcCCAGGGGTTCTGGTCCTCA | |  | | |
| Note：The lowercase letters represent the homologous recombination and restriction enzyme sites, while the uppercase letters indicate the target gene sequence. | | | | | | | | |
| Table S2 *Pi65* screened the genes of yeast library | | | | | |  |  |  |
| Serial | Gene ID | | | Annotation | | No. |  |  |
| 1 | LOC_Os03g29810 | | | OsClp6 - Putative Clp protease homologue | | 31 |  |  |
| 2 | LOC_Os04g56430 | | | cysteine-rich receptor-like protein kinase, putative, expressed Length: 777 | | 13 |  |  |
| 3 | LOC_Os08g44680 | | | photosystem I reaction center subunit II, chloroplast precursor | | 12 |  |  |
| 4 | LOC_Os08g43560 | | | OsAPx4 - Peroxisomal Ascorbate Peroxidase encoding gene 5,8,9 | | 7 |  |  |
| 5 | LOC_Os01g64960 | | | chlorophyll A-B binding protein, putative, expressed Length: 1480 | | 5 |  |  |
| 6 | LOC_Os06g22960 | | | aquaporin protein, putative, expressed Length: 747 | | 4 |  |  |
| 7 | LOC_Os01g58890 | | | proteinase inhibitor precursor protein, putative, expressed Length: 423 | | 3 |  |  |
| 8 | LOC_Os12g19381 | | | ribulose bisphosphate carboxylase small chain, chloroplast precursor | | 3 |  |  |
| 9 | LOC_Os03g20370 | | | Calmodulin, Ca<sup>2+</sup> sensor, signalling, Thermotolerance | | 3 |  |  |
| 10 | LOC_Os12g24020 | | | rhodanese-like domain containing protein, putative, expressed | | 3 |  |  |
| 11 | LOC_Os01g54930 | | | one zinc finger protein | | 2 |  |  |
| 12 | LOC_Os05g43950 | | | vascular plant one zinc finger protein 2 | | 2 |  |  |
| 13 | LOC_Os01g03340 | | | BBTI4 - Bowman-Birk type bran trypsin inhibitor precursor | | 2 |  |  |
| 14 | LOC_Os01g19740 | | | calvin cycle protein CP12, putative, expressed Length: 375 | | 2 |  |  |
| 15 | LOC_Os03g38730 | | | peroxisomal membrane protein, putative, expressed Length: 1077 | | 2 |  |  |
| 16 | LOC_Os10g38910 | | | expressed protein Length: 666 Number of Matches: 1 | | 2 |  |  |
| 17 | LOC_Os01g59080 | | | expressed protein Length: 630 Number of Matches: 1 | | 2 |  |  |
| 18 | LOC_Os03g24580 | | | transmembrane protein 85 | | 2 |  |  |
| 19 | LOC_Os04g33940 | | | protein of unknown function DUF1279 domain containing protein | | 2 |  |  |
| 20 | LOC_Os07g05810 | | | glycine-rich protein, putative, expressed Length: 843 Number of Matches: 1 | | 2 |  |  |
| 21 | LOC_Os07g09000 | | | WD domain, G-beta repeat domain containing protein | | 2 |  |  |
| 22 | LOC_Os01g45274 | | | carbonic anhydrase, chloroplast precursor, putative, expressed | | 2 |  |  |
| 23 | LOC_Os09g24590 | | | expressed protein | | 2 |  |  |
| 24 | LOC_Os07g36140 | | | core histone H2A/H2B/H3/H4, putative, expressed | | 2 |  |  |
| 25 | LOC_Os03g55030 | | | UDP-glucoronosyl and UDP-glucosyl transferase domain containing protein | | 2 |  |  |
| 26 | LOC_Os01g17170 | | | magnesium-protoporphyrin IX monomethyl ester cyclase,chloroplast precursor | | 1 |  |  |
| 27 | LOC_Os01g31690 | | | oxygen-evolving enhancer protein 1, chloroplast precurso | | 1 |  |  |
| 28 | LOC_Os01g42430 | | | vacuolar ATP synthase, putative | | 1 |  |  |
| 29 | LOC_Os01g49030 | | | expressed protein Length: 759 Number of Matches: 1 | | 1 |  |  |
| 30 | LOC_Os01g55830 | | | glutathione S-transferase, putative | | 1 |  |  |
| 31 | LOC_Os01g56680 | | | photosystem II reaction center W protein, chloroplast precursor | | 1 |  |  |
| 32 | LOC_Os01g58890 | | | cysteine proteinase inhibitor precursor protein, putative | | 1 |  |  |
| 33 | LOC_Os01g68480 | | | thioredoxin, putative | | 1 |  |  |
| 34 | LOC_Os02g02720 | | | SNARE domain containing protein, putative | | 1 |  |  |
| 35 | LOC_Os02g08180 | | | protein transport protein SEC61 subunit gamma | | 1 |  |  |
| 36 | LOC_Os02g32200 | | | thioesterase family protein, putative | | 1 |  |  |
| 37 | LOC_Os02g44290 | | | phosphatase, putative, expressed Length: 618 Number of Matches: 1 | | 1 |  |  |
| 38 | LOC_Os03g03720 | | | glyceraldehyde-3-phosphate dehydrogenase | | 1 |  |  |
| 39 | LOC_Os03g17010 | | | RNA recognition motif containing protein, putative | | 1 |  |  |
| 40 | LOC_Os03g18690 | | | 26S protease regulatory subunit 4, putative | | 1 |  |  |
| 41 | LOC_Os03g31410 | | | expressed protein Length: 258 Number of Matches: 1 | | 1 |  |  |
| 42 | LOC_Os04g10240 | | | nicotiana lesion-inducing like | | 1 |  |  |
| 43 | LOC_Os04g48530 | | | C4-dicarboxylate transporter/malic acid transport protein | | 1 |  |  |
| 44 | LOC_Os04g54630 | | | expressed protein Length: 585 Number of Matches: 1 | | 1 |  |  |
| 45 | LOC_Os05g03140 | | | tetraspanin family protein, putative | | 1 |  |  |
| 46 | LOC_Os05g07700 | | | ribosomal protein, putative | | 1 |  |  |
| 47 | LOC_Os05g27010 | | | peptide transporter PTR3-A | | 1 |  |  |
| 48 | LOC_Os05g41210 | | | OsCam2 - Calmodulin | | 1 |  |  |
| 49 | LOC_Os05g47470 | | | VIP1 protein, putative | | 1 |  |  |
| 50 | LOC_Os05g51180 | | | hyaluronan/mRNA binding family domain containing protein | | 1 |  |  |
| 51 | LOC_Os06g01850 | | | ferredoxin--NADP reductase, chloroplast precursor, putative | | 1 |  |  |
| 52 | LOC_Os06g21590 | | | chlorophyll A-B binding protein, putative | | 1 |  |  |
| 53 | LOC_Os07g03730 | | | SCP-like extracellular protein, expressed Length | | 1 |  |  |
| 54 | LOC_Os07g05580 | | | ribosomal protein L7Ae, putative, expressed Length | | 1 |  |  |
| 55 | LOC_Os07g06660 | | | glyoxalase family protein, putative, expressed Length | | 1 |  |  |
| 56 | LOC_Os08g06530 | | | rubredoxin family protein, putative, expressed Length | | 1 |  |  |
| 57 | LOC_Os08g39630 | | | helix-loop-helix DNA-binding domain containing protein | | 1 |  |  |
| 58 | LOC_Os09g11520 | | | DUF565 domain containing protein, putative | | 1 |  |  |
| 59 | LOC_Os09g27690 | | | expressed protein Length: 714 Number of Matches: 1 | | 1 |  |  |
| 60 | LOC_Os09g36710 | | | peptidase, T1 family, putative, expressed Length | | 1 |  |  |
| 61 | LOC_Os10g19970 | | | expressed protein Length: 441 Number of Matches: 3 | | 1 |  |  |
| 62 | LOC_Os11g06890 | | | vacuolar ATP synthase, putative, expressed Length | | 1 |  |  |
| 63 | LOC_Os12g17830 | | | expressed protein Length: 904 Number of Matches: 1 | | 1 |  |  |
| 64 | LOC_Os12g24020 | | | rhodanese-like domain containing protein | | 1 |  |  |
| 65 | LOC_Os12g32180 | | | cornichon protein, putative, expressed Length | | 1 |  |  |
| 66 | LOC_Os01g03390 | | | BBTI7 - Bowman-Birk type bran trypsin inhibitor precursor, expressed | | 1 |  |  |
| 67 | LOC_Os02g44930 | | | HMG1/2, putative, expressed | | 1 |  |  |
| 68 | LOC_Os11g47970 | | | AAA-type ATPase family protein, putative, expressed | | 1 |  |  |
| 69 | LOC_Os01g13690 | | | ligA, putative, expressed | | 1 |  |  |
| 70 | LOC_Os12g42090 | | | methyltransferase domain containing protein, putative, expressed | | 1 |  |  |
| 71 | LOC_Os12g08730 | | | thioredoxin, putative, expressed | | 1 |  |  |
| 72 | LOC_Os01g23740 | | | OsPDIL2-2 protein disulfide isomerase PDIL2-2, expressed | | 1 |  |  |
| 73 | LOC_Os05g01050 | | | DNA-binding protein-related, putative, expressed | | 1 |  |  |
| 74 | LOC_Os10g35630 | | | cystathionin beta synthase protein, putative, expressed | | 1 |  |  |
| 75 | LOC_Os05g28740 | | | universal stress protein domain containing protein | | 1 |  |  |
| 76 | LOC_Os06g01210 | | | Plastocyanin, chloroplast precursor. | | 1 |  |  |
| 77 | LOC_Os04g46880 | | | transporter, major facilitator family, putative, expressed | | 1 |  |  |
| 78 | LOC_Os01g37850 | | | expressed protein | | 1 |  |  |
| 79 | LOC_Os04g24140 | | | ribose-5-phosphate isomerase A, putative, expressed | | 1 |  |  |
| 80 | LOC_Os08g44640 | | | OsSCP41 - Putative Serine Carboxypeptidase homologue | | 1 |  |  |
| 81 | LOC_Os05g34680 | | | expressed protein | | 1 |  |  |
| 82 | LOC_Os05g45660 | | | plasminogen activator inhibitor 1 RNA-binding protein | | 1 |  |  |
| 83 | LOC_Os02g50174 | | | caleosin related protein, putative, expressed | | 1 |  |  |
| 84 | LOC_Os02g02400 | | | Similar to Catalase. | | 1 |  |  |
| 85 | LOC_Os03g07300 | | | ribulose-phosphate 3-epimerase, chloroplast precursor | | 1 |  |  |
| 86 | LOC_Os03g63950 | | | plastid-specific 30S ribosomal protein 1, chloroplast precursor | | 1 |  |  |
| 87 | LOC_Os07g03200 | | | Similar to Phytosulfokines 4 precursor. | | 1 |  |  |
| 88 | LOC_Os10g39120 | | | ubiquitin-conjugating enzyme, putative, expressed | | 1 |  |  |
| 89 | LOC_Os06g49210 | | | retrotransposon protein, putative, unclassified, expressed | | 1 |  |  |
| 90 | LOC_Os03g62010 | | | harpin-induced protein 1 domain containing protein, expressed | | 1 |  |  |
| 91 | LOC_Os03g15960 | | | hsp20/alpha crystallin family protein, putative, expressed | | 1 |  |  |
| 92 | LOC_Os03g49450 | | | expressed protein | | 1 |  |  |
| 93 | LOC_Os03g05520 | | | nicotiana lesion-inducing like, putative, expressed | | 1 |  |  |
| 94 | LOC_Os09g24924 | | | transporter family protein, putative, expressed | | 1 |  |  |
| 95 | LOC_Os07g36410 | | | expressed protein | | 1 |  |  |
| 96 | LOC_Os09g37910 | | | HMG1/2, putative, expressed | | 1 |  |  |
| 97 | LOC_Os02g42320 | | | peptidase, T1 family, putative, expressed | | 1 |  |  |
| 98 | LOC_Os07g12650 | | | ribosomal protein L7Ae, putative, expressed | | 1 |  |  |
| 99 | LOC_Os03g08050 | | | elongation factor Tu, putative, expressed | | 1 |  |  |
| 100 | LOC_Os05g41060 | | | ADP-ribosylation factor, putative, expressed | | 1 |  |  |
