# Supplementary material for: Protein kinase Pi65 regulates rice blast resistance through phosphorylation-dependent signaling and metabolic reprogramming
Source: Front Genet. 2026 Jan 5;16:1715247. doi: 10.3389/fgene.2025.1715247 (PMC12812394; doi:10.3389/fgene.2025.1715247)
Supplement: Supplementary file 2 [file DataSheet1.docx]

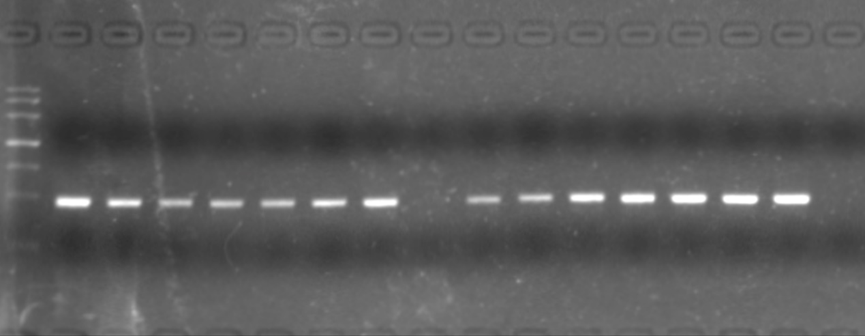


**Supplementary Figure 1.** Detection of hygromycin in transgenic lines. Note: Lane 1, DL2000Plus; Lanes 2-15, detection of hygromycin in transgenic lines; Lane 16, ddH_2_O negative control.


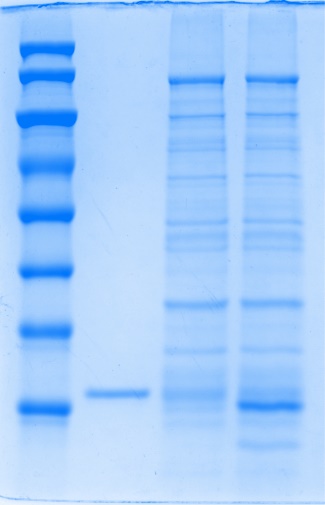


**Supplementary Figure 2.** The original image for Figure 1A.


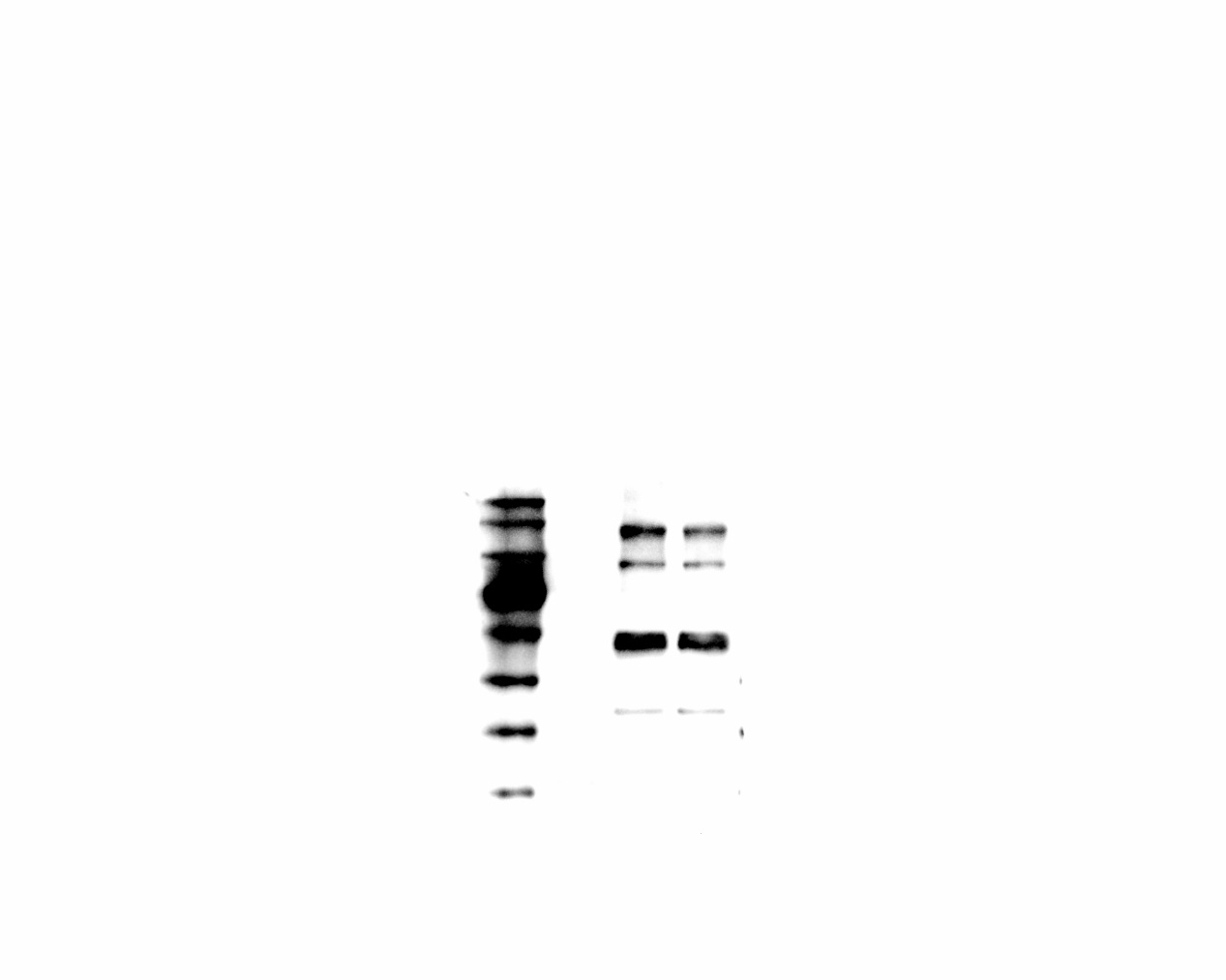


**Supplementary Figure 3.** The original image for Figure 1B.


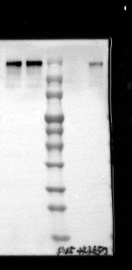


**Supplementary Figure 4.** The original image for Figure 5C Anti-HA.


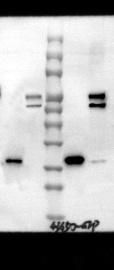


**Supplementary Figure 5.** The original image for Figure 5C Anti-GFP.


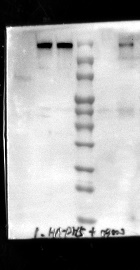


**Supplementary Figure 6.** The original image for Figure 6C Anti-HA.


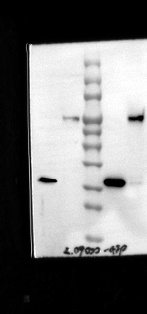


**Supplementary Figure 7.** The original image for Figure 6C Anti-GFP.
